# Unbiasedly decoding the tumor microenvironment with single-cell multiomics analysis in pancreatic cancer

**DOI:** 10.1186/s12943-024-02050-7

**Published:** 2024-07-09

**Authors:** Yifan Fu, Jinxin Tao, Tao Liu, Yueze Liu, Jiangdong Qiu, Dan Su, Ruobing Wang, Wenhao Luo, Zhe Cao, Guihu Weng, Taiping Zhang, Yupei Zhao

**Affiliations:** 1 General Surgery Department, State Key Laboratory of Complex Severe and Rare Diseases, Peking Union Medical College Hospital, Chinese Academy of Medical Sciences and Peking Union Medical College, Beijing, 100730 China; 2 4+4 Medical Doctor Program, Chinese Academy of Medical Sciences and Peking Union Medical College, Beijing, 100730 China; 3 Clinical Immunology Center, Chinese Academy of Medical Sciences and Peking Union Medical College, Beijing, 100730 China

**Keywords:** Pancreatic cancer, Single-cell, Multiomics, Tumor microenvironment

## Abstract

Pancreatic ductal adenocarcinoma (PDAC) is a highly aggressive malignancy with a poor prognosis and limited therapeutic options. Research on the tumor microenvironment (TME) of PDAC has propelled the development of immunotherapeutic and targeted therapeutic strategies with a promising future. The emergence of single-cell sequencing and mass spectrometry technologies, coupled with spatial omics, has collectively revealed the heterogeneity of the TME from a multiomics perspective, outlined the development trajectories of cell lineages, and revealed important functions of previously underrated myeloid cells and tumor stroma cells. Concurrently, these findings necessitated more refined annotations of biological functions at the cell cluster or single-cell level. Precise identification of all cell clusters is urgently needed to determine whether they have been investigated adequately and to identify target cell clusters with antitumor potential, design compatible treatment strategies, and determine treatment resistance. Here, we summarize recent research on the PDAC TME at the single-cell multiomics level, with an unbiased focus on the functions and potential classification bases of every cellular component within the TME, and look forward to the prospects of integrating single-cell multiomics data and retrospectively reusing bulk sequencing data, hoping to provide new insights into the PDAC TME.

## Research on the PDAC TME: entering the single-cell multiomics era

Pancreatic ductal adenocarcinoma (PDAC) is a highly aggressive malignant tumor characterized by a dismal prognosis, limited treatment response, and late-stage diagnosis [1]. Sequencing and mass spectrometry techniques have long been used for the discovery of the PDAC tumor microenvironment (TME), from the genome to the transcriptome and metabolome, and from bulk sequencing to the single-cell level, which has gradually revealed the intrinsic and vital heterogeneity in the TME.

Here, we comprehensively review how multiomics analyses at the single-cell level contribute to our understanding of the PDAC TME, including reports of rare cell types, advancements in the study of the heterogeneity of each cellular component, and the development of targeted and sensitization therapeutic strategies for PDAC. We also focus on the marker genes of PDAC cell components within the TME and their relationships with biological functions, as well as their impacts on the initiation, progression, and metastasis of PDAC. Simultaneously, we summarize the latest methods and software packages for fully leveraging non-single-cell data, aiming to ultimately provide definitive single-cell insights into the PDAC TME.

## Genome-to-multiomics sequencing of PDAC

Initially, at the genomic level, multiple pancreatic cancer subtypes, of which PDAC comprises the largest share, have been subjected to numerous landscape-oriented studies [2–4]. These investigations have comprehensively delineated the genomic instability and copy number variations of PDAC while also elucidating common genetic mutations, such as those in KRAS, TP53, and SMAD4. Notably, these mutations are heterogeneous among patients from different countries; for example, patients with core DNA damage response gene mutations and patients carrying TP53 mutations are mutually exclusive [3, 5]. In addition, single-cell genome sequencing has been widely used to study the development of tumors because stable monoclonal sources of tumor cells always exhibit similar genome patterns, including mutations and copy number variations (CNVs). Recent opinion is that, a tumor was proposed to encompass cells from independent multiclonal origins, yet only one may grow to a detectable size [6]. Hence, single-cell genomics sequencing is useful for identifying malignant clonal cell clusters. Microsatellite instability is also investigated in PDAC tumor cells, but this mechanism is common in colorectal cancer [7, 8]. At the transcriptomic level, in contrast to genomic research, rapid advancements in next-generation sequencing (NGS) and single-cell/single-nuclei RNA sequencing (sc/snRNA-seq) technologies have led to the emergence of a significant number of atlas-type studies [9–12]. Henceforth, PDAC research has advanced to the single-cell dimension, enabling researchers to identify various cellular components within complex tumor tissues, such as malignant tumor cells, tumor stromal cells, and immune cells. These findings have greatly enhanced our understanding of the heterogeneity within the TME. Moreover, the scope of transcriptomic research has expanded to include the regulation of the entire transcriptional process, from transcription initiation to epigenetic modifications [13, 14]. These single-cell transcriptomic studies also encompass multiple aspects of tumor biology, including initiation [15], progression [16], and metastasis with patient specimens [11], patient-derived organoids [17], patient-derived xenografts [18], mouse models with cell lines (BxPC-3, PANC-1, ASPC-1, Mia PaCa-2, etc.) [19, 20].

Beyond the transcriptomic level, the emergence of single-cell proteomics and metabolomics technologies based on single-cell mass spectra is promising for exploring the tumor microenvironment [21]. However, due to technical limitations, single-cell proteomics and metabolomics have not yet been widely applied. Currently, several alternative solutions have emerged for the methodological development of single-cell proteomics, which will be discussed in detail later in the text.

In addition, time and space represent additional dimensions in the study of the TME of PDAC. The advent of spatial transcriptomics has increased the focus on the distribution of various cells within PDAC, providing spatial annotations for single-cell transcriptomic studies. This technology not only supports traditional analyses of intercellular interactions based on expression patterns, but also achieve cellular or even subcellular spatial precision with the latest advancements. These developments have significantly enhanced our understanding of the cellular components within the PDAC TME and have led to novel targeted therapeutic strategies [22]. However, scRNA-seq has remained absolutely central in recent studies on PDAC.

## Primary catalog of single-cell analyses of the TME

In the workflow of scRNA-seq analysis, annotating and classifying cell clusters play crucial roles in all downstream analyses. To date, numerous annotation strategies for scRNA-seq data have been developed based on gene markers/signatures, transfer learning using external references (SingleR [23] and Celltypist [24, 25]), and semisupervised annotation (SCINA [26]), and even been utilized with the assistance of ChatGPT [27, 28]. The SingleR package is one of the most popular tools; however, recent studies have shown that Celltypist might deliver more accurate annotation results [25]. Nevertheless, automated tools face limitations from pretrained models or input external references, potentially introducing biases, especially in certain contexts such as the PDAC TME. Most of the recent studies we reviewed still preferred gene markers for accurate determination.

Hence, a clear understanding of the composition and marker genes of each subpopulation is vitally important, as scRNA-seq technology frequently encounters the dropout phenomenon, which means that genes are not expressed in certain cells but are highly expressed in other cells. The percentage of dropouts in single-cell transcriptome data can reach 50%, which might be caused by the library construction process [29]. Generally, two methods have been developed to solve this problem: imputation and dimensionality reduction. The original version of Seurat was developed and used to map the scRNA-seq data with in situ RNA patterns [30]. As most researchers choose Seaurat or scanpy for scRNA-seq analysis, the dimensionality reduction, clustering, and annotation of cell clusters have formed a conventional pipeline. Here, we will not discuss the computational and mathematical theory, which is reviewed in other studies [31–34]. Briefly, this pipeline always reduces the high-dimensional data and calculates the principal components (PCs), and the reduction is applied based on the PCs rather than a single gene. With this method, the annotation of cells is not significantly affected by the loss of simple genes in a single cell. Moreover, recent advanced sequencing methods can capture approximately 10,000 cells per sample, and increasing the number of captured cells will also decrease the imperfection of gene dropout.

The same issue also exists in single-cell proteomics and metabolomics data, but most researchers removed all dropout data or imputed zeros or random values [35, 36]. We believe that the above methods could contribute to these omics analyses because of the use of similar abundance or expression matrix data.

**Fig. 1:**
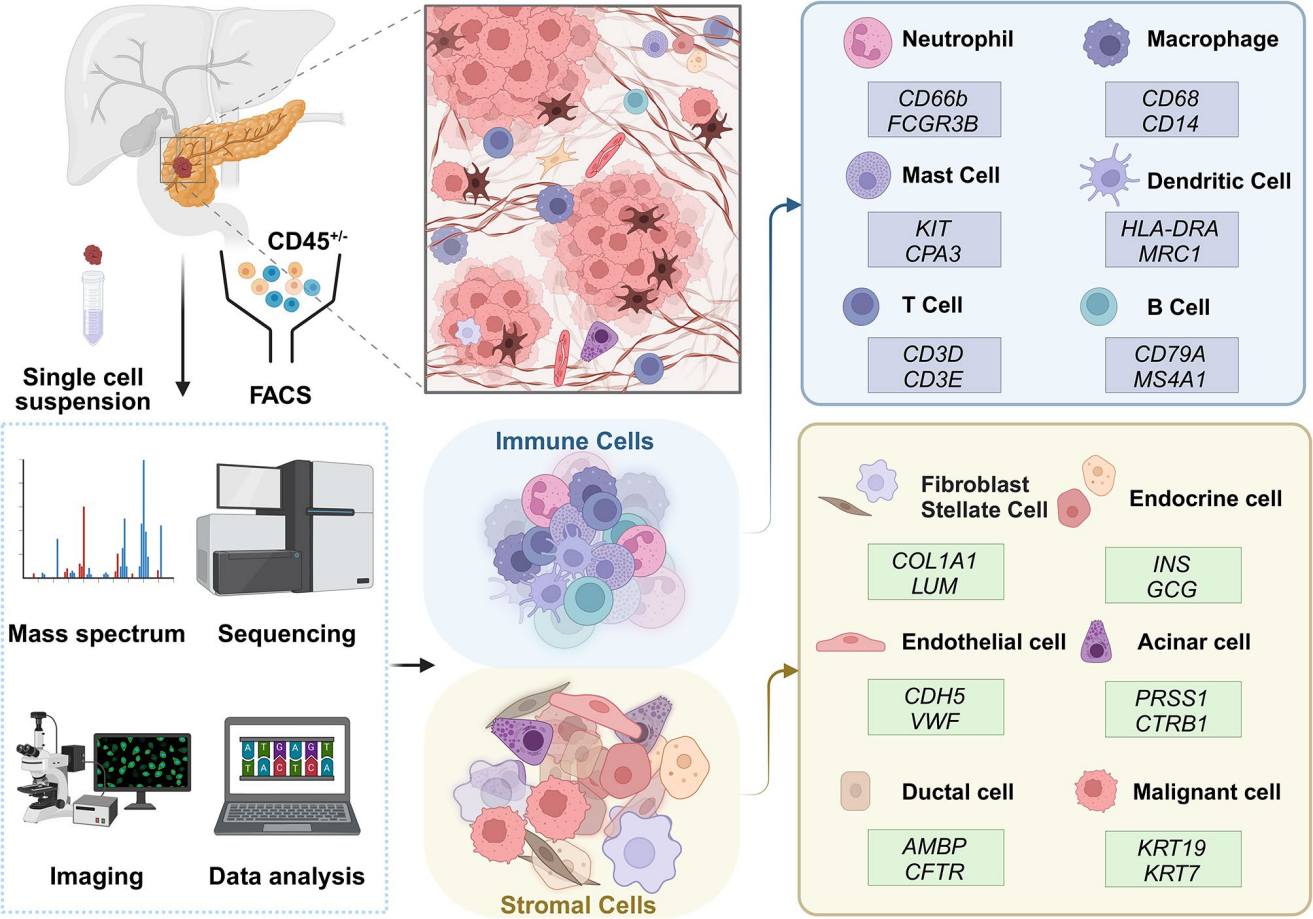
Single-cell profiling strategies and landscape of the PDAC TME. The single-cell analysis pipeline involves dissociation of PDAC tumors, cell sorting by fluorescence-activated cell sorting (FACS), mass cytometry or sequencing, and data integration. Two main cell types of the PDAC TME were defined by their canonical marker genes

Concurrently, many studies have defined many cell clusters and their marker genes at various levels of distinction [10, 19, 37]. Most researchers often face challenges in robustly mapping their cell clusters with the extant literature. The data are confusing when the authors attempt to use signatures instead of specific gene markers or when the specificity of marker genes is weak. Moreover, numerous studies have been conducted on various components of the PDAC TME, with a primary focus on immune cells, malignant epithelial cells, and cancer associated fibroblasts (CAFs). We first summarize the canonical gene markers of the stroma and immune system in the PDAC TME (Fig. 1). However, despite fewer studies, substantial evidence indicates that other components, including endocrine cells, acinar cells, endothelial cells, and mast cells, also play significant roles in the onset and progression of PDAC.

## Decoding immune components in the TME: a promising and effective strategy

**Fig. 2:**
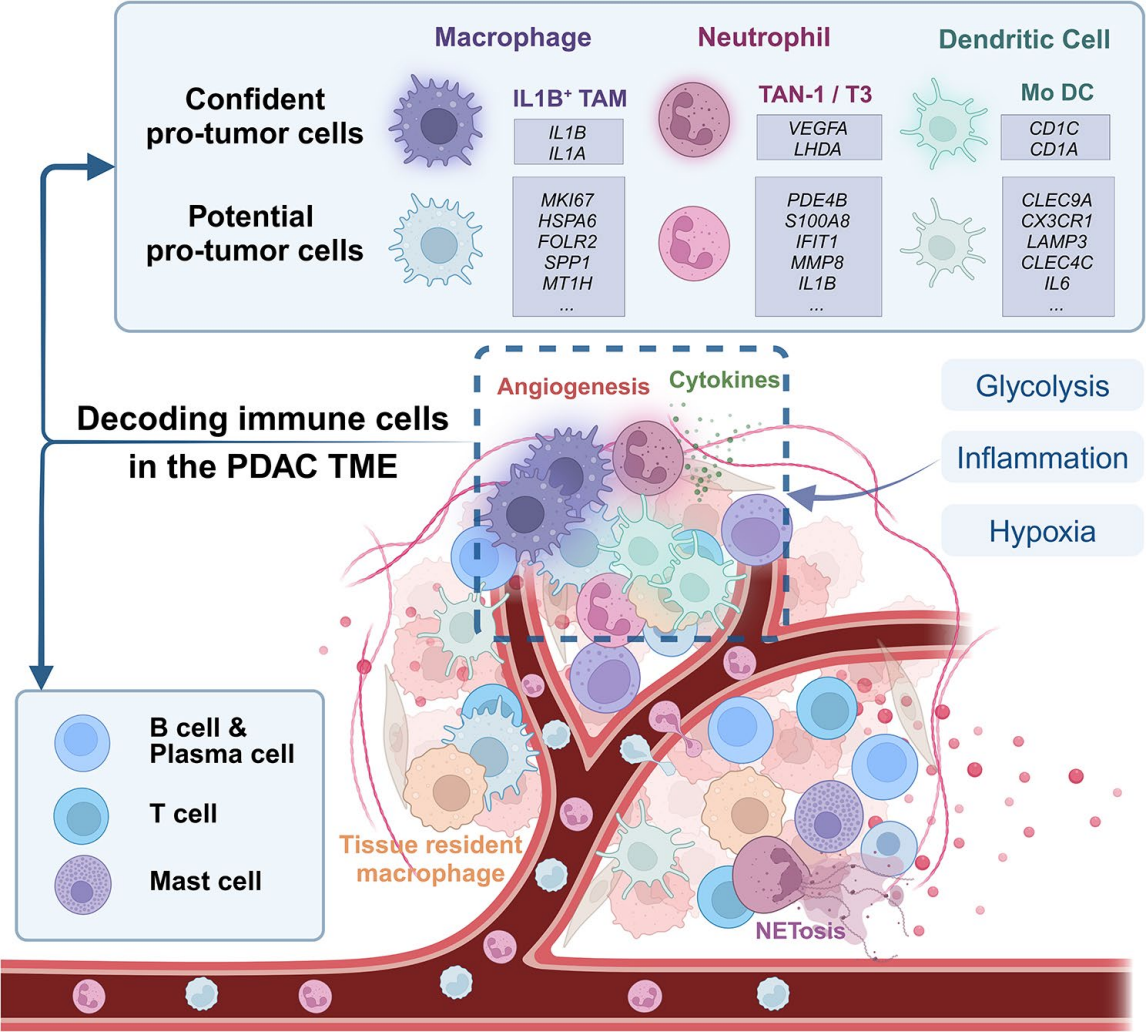
Landscape of immune cells. Myeloid cells that confidently promote tumor were highlighted with marker genes. Neutrophils and macrophages were divided into tissue-resident type and peripheral circulation type. The focal niche was characterized by the activation of glycolysis, inflammation and hypoxia together with angiogenesis and secretion of cytokines

The immune cells in the PDAC TME include various lymphocytes and myeloid cell components that collectively confer the immunosuppressive characteristics of the PDAC TME. Traditionally, myeloid cells in the TME are primarily considered to include tumor-associated macrophages (TAMs) or myeloid-derived suppressor cells (MDSCs), which are marked by S100A8, S100A9, and S100A12 [37, 38]. These components are considered to be strongly associated with low infiltration of CD8^+^ T cells and the induction of an immunosuppressive TME [37]. CRIP1 expressed in tumor cells promotes the infiltration of MDSCs via CXCL1/5-CXCR1/2 signaling, and the inhibition of CXCR1/2 decreases the recruitment of MDSCs and enhances the antitumor effect of PD-L1 treatment to improve resistance to immune-checkpoint blockers(ICBs) [37]. Another combination of anti-CXCR2 therapy and immunotherapy was designed to target CXCR2, 41BB, and LAG3, and this strategy was shown to increase antitumor T cell infiltration, increase the diversity of T cells, decrease MDSC infiltration, and reprogram the immune components of the PDAC TME [39]. However, the use of cell surface markers for sorting cells presents challenges, as polymorphonuclear myeloid-derived suppressor cells (PMN-MDSCs) are phenotypically and morphologically similar to neutrophils. Overlapping molecular markers also make distinguishing between PMN-MDSCs and neutrophils difficult using common research methods. With scRNA-seq, MDSCs are categorized as neutrophils, mast cells, and dendritic cells based on their expression profiles of various expression patterns, providing compelling evidence [40]. Studies have also documented the preferential spatial arrangement of immune cells in the PDAC TME [41]. Here, we mainly discuss how single-cell sequencing contributes to identifying each myeloid component (Fig. 2).

## Macrophages respond to alterations in metabolism, inflammation, and stromal collagen

Macrophages, as key components of innate immunity, perform multiple functions, including phagocytosis, immune activation/suppression, metabolic regulation, growth support, angiogenesis induction, and evasion support [42]. Different macrophages can either maintain or disrupt the homeostasis of the TME, are highly heterogeneous in their impacts on tumors, and influenced by intrinsic factors such as genetics, immunity, and environmental signals [43]. In the PDAC TME, macrophages are a core focus of many immunotherapeutic strategies. Most views suggest that macrophage infiltration in PDAC is associated with a poor prognosis, and many relevant predictive markers have been identified [44, 45].

Single-cell sequencing revealed that TAMs in the PDAC TME play a key role in regulating the composition of the tumor matrix. On the one hand, they can directly produce collagen or engage in collagen endocytosis through a mechanism dependent on the mannose receptor (MRC1), and on the other hand, they can indirectly regulate collagen synthesis and breakdown through interactions with CAFs and/or tumor cells [46, 47]. The number of MRC1^+^ TAMs decreased after KRAS inhibition, while the number of CD11b^+^F4/80^+^ macrophages remained stable [48]. CAFs can also drive monocyte differentiation into immunosuppressive TAMs through interactions between sialic acid and Siglec receptors [49]. TAM functions are also modulated by neuronal signals, thereby promoting PDAC progression [50].

The suppression of macrophages revealed by scRNA-seq has been shown to alleviate weight loss in PDAC mice, delay muscle wasting, and mitigate the occurrence and development of cachexia in PDAC mice. This conclusion has also been confirmed in PDAC patients, with macrophage infiltration positively correlated with the expression levels of the inflammatory factor TWEAK. Interestingly, while TWEAK secretion is not high in PDAC tumor cells, macrophages can respond to tumor cell-secreted CCL2, activate and secrete CCL5, and significantly upregulate TWEAK expression and secretion in tumor cells via the TRAF6/NF-κB pathway [51]. Simply suppressing macrophages may also cause a compensatory recruitment of granulocytes and monocytes, thereby weakening the therapeutic effect. Therefore, some studies have used a CD11b agonist to inhibit the NF-κB/IL1 signaling pathway, reduce macrophage infiltration, and activate the CD11b/FAK/ROS/STING/IFN signaling pathway to increase CD8^+^ T cell infiltration and produce an antitumor immune effect that improves the prognosis of PDAC patients [44]. Galectin-3 expression is associated with macrophage infiltration in the PDAC TME and PDAC patient survival, and is concentrated in regions of tumors that are enriched with cancer cells [52]. Galectin-3 knockout or inhibition can enhance the efficacy of anti-PD-1 and anti-CXCL12/CXCR4 therapies [52]. Deoxycytidine blockade of TAMs impairs the cytotoxic activity of gemcitabine in PDAC tumor cells, and activation of the CCL5/CCR5/Sp1/CD44 signaling pathway in TAMs also inhibits the antitumor efficacy of gemcitabine. Inhibiting TAM recruitment may also sensitize patients to adjuvant therapy [53, 54].

Single-cell studies have provided new insights into the subtypes of TAMs in the PDAC TME. Fortunately, these TAM subtypes are relatively unified and possess stable biological characteristics to some extent. Recent studies have classified TAMs from PDAC patients using markers such as MKI67, IL1B, HSP, FOLR2, SPP1, and MT1H [19]. A mouse model was generated, and the similarity between mouse Il1b^+^ macrophages and human IL1B^+^ TAMs was verified by transfer annotation. Due to the distinct physiological functions of tissue-resident macrophages and peripherally derived monocytes that differentiate into macrophages [55], this study specifically highlights that IL1B^+^ TAMs are derived from monocyte and are active in biological functions such as the inflammatory response, leukocyte recruitment, and angiogenesis (Table 1) [19].

The developmental trajectory of IL1B^+^ TAMs has also been delineated; stimulation with IL-1 and TNF leads to the differentiation of monocytes into IL1B^+^ TAMs, which are primarily located around the tumor core in the matrix rich in CAFs and close to hypoxic areas within the tumor. By targeting the IL-1β-mediated inflammatory response during this process, the COX2 inhibitor celecoxib can significantly inhibit PDAC progression through an interferon-independent mechanism. Moreover, IL-1β signaling has been shown to interact with IL1B^+^ TAMs in tumor cells, creating a positive feedback loop that continuously promotes PDAC progression.

Several studies have also provided evidence for identifying macrophages at spatial resolution. Stress response genes were used to localize to specific sites in the PDAC TME, and monocytes and macrophages were found to be enriched in these areas [41]. Il-1β^+^ TAMs were detected in the core of tumors surrounded by CAFs and were enriched in areas characterized by inflammation, hypoxia, and angiogenesis [19]. CD163^+^ macrophages were found to be localized to PRF1^+^CD8^+^ T cells, which express perforin and are mostly found at the border of tumors [56]. These results were confirmed by immunofluorescence staining.

Methods targeting specific TAMs subtypes based on TAMs subtyping have been developed and proven effective. For example, through the action of progranulin on CFTR, macrophages with efferocytosis function can undergo lysosomal acidification mediated by LXRα, transforming macrophages into an immunosuppressive phenotype and upregulating the T cell suppressor gene Arg1 [57]. Targeting this group of TAMs with a Mer tyrosine kinase inhibitor can block their efferocytosis function, enhance the activity of CD8^+^ T cells, restore antitumor immune function in PDAC liver metastasis, and prevent tumor metastasis and growth [57]. Other evidence also suggested that specific subtypes of macrophages could educate terminally exhausted T cells to acquire a regulatory phenotype [56].

**Table 1:** The markers and functions of macrophage subclusters identified in the PDAC TME

Macrophage Types	Markers	Functions
Pan-Macrophage [9, 16]	CD68, CD14	
M1-Macrophage [58]	TLR2, TLR4, CD80, CD86, iNOS, TNFA, IL1B, IL6, CXCL9	Pro-inflammation
M2-Macrophage [58]	CD206, CD163, CD209, FIZZ1, IL10, TGFB1, CCL1, CCL17, ARG1	Anti-inflammation
MKI67^+^ TAM [19]	MKI67, TOP2A, PCLAF, UBE2C, TK1	Expressed cell cycle genes
IL1B^+^ TAM [19]	IL1B, IL1A, NLRP3, PTGS2, CCL3	Inflammatory response, leukocyte recruitment and angiogenesis
HSP^+^ TAM [19]	HSPA6, SERPINH1, BAG3, HSPB1, HSPD1	Expressed heat-shock protein
FOLR2^+^ TAM [19]	FOLR2, LYVE1, SELENOP, SLC40A1, MRC1	Non-canonical myeloid marker, and matched resident macrophages
SPP1^+^ TAM [19, 38]	SPP1, MARCO, FBP1, APOC1, LIPA	Related to lipid metabolism and expressed phagocytic receptor
MT^+^ TAM [19]	MT1H, MT1G, MT1X, MT1E, MT2A	Expressed metallothionein

## Neutrophil migration trajectories and acquired characteristics

Studies of neutrophils, particularly within the PDAC TME, are limited by sequencing technology and the short lifetime of neutrophils, lagging behind other immune components [59]. Prompt collection of patient samples and the selection of appropriate sequencing methods are crucial to capture a sufficient number of neutrophils for downstream analyses; the popular 10x sequencing platform appears to underperform in this regard.

### The ability of neutrophils to predict the onset, progression, prognosis, metastasis, and recurrence of pancreatic cancer has been increasingly demonstrated, largely through the machine learning models and novel algorithms

Initially, capturing neutrophils with RNA-seq was difficult, and researchers used immunofluorescence staining to identify neutrophils in PDAC. Limited by the low throughput and the resolution of immunofluorescence staining, this method only roughly assesses the relative infiltration of neutrophils and characterizes the classical N1 and N2 phenotypes [60]. In patients who underwent radical resection for PDAC, the quantity of neutrophils correlated significantly with the infiltration levels of CD8^+^ T cells and Treg cells [61]. Notably, the numbers of N2 neutrophils were significantly increased compared to N1 neutrophils, and patients with a higher N1/N2 ratio experienced longer overall survival and recurrence-free survival, with relatively favorable tumor differentiation, lymph node metastasis, and TNM staging.

### Previous studies without high-throughput sequencing have shown that CXCL1 and CXCL2 signals are crucial chemotactic signals for neutrophil function in PDAC

Regarding epigenetic regulation, a lack of SETD2 promotes immune evasion by PDAC tumor cells, with neutrophils undergoing the most significant reprogramming toward an immunosuppressive phenotype [62]. A possible mechanism is that the loss of SETD2-H3K36me3 leads to an aberrant increase in H3K27me3 levels, which downregulates CXADR expression. The overexpression of CXCL1 and GM-CSF via the PI3K-AKT pathway and the recruitment and reprogramming of neutrophils suppress the cytotoxicity of CD8^+^ T cells, promoting tumor progression [63]. Studies have shown that neutrophils also significantly impact PDAC liver metastasis. After chemotherapy, neutrophils are recruited via tumor cell-secreted CXCL1 and CXCL2 signaling to the liver, where chemotherapy increases the expression of growth arrest specific 6 (Gas6) in circulating neutrophils. These neutrophils then recognize the AXL receptor on liver metastatic tumor cells, thereby promoting the growth of liver metastatic tumor cells [64]. However, in contrast to the traditional view that neutrophils affect the infiltration and activation of CD8^+^ T cells, depleting neutrophils during gemcitabine treatment did not significantly affect CD8^+^ T cells, suggesting that neutrophils promote PDAC metastatic recurrence in a CD8^+^ T cell-independent manner [64].

### Neutrophils within the tumor also play a crucial role

In the PDAC TME, neutrophils are primarily recruited and mature from their precursor cells in the circulatory system [65]. In mouse models of PDAC, supplementation with melatonin can specifically increase the number of CD11b^+^Ly6G^+^ neutrophils that with high TNFα activity within the tumor, killing tumor cells via direct cell-to-cell contact mechanisms and thereby inhibiting tumor growth. However, melatonin-induced neutrophils exhibit unique immunological characteristics, such as being recruited to the tumor microenvironment predominantly by tumor cell-released CXCL2, rather than by chemokines secreted by macrophages, to induce reactive oxygen species (ROS)-dependent formation of neutrophil extracellular traps (NETs) that kill tumor cells. Interestingly, these neutrophils do not inhibit the cytotoxic activity or proliferative capacity of CD8^+^ T cells, suggesting that NETosis might have predictive value for the outcomes of patients with PDAC [66].

The collective evidence from multiple studies indicates substantial heterogeneity within neutrophils, far exceeding the simple N1 and N2 classification. Different neutrophil subsets can respond to various signals, exerting stimulatory, inhibitory, or unique biological functions independent of certain immune components within the TME. In addition to classification, knockout mouse models have been used to specifically investigate the function of neutrophils in pancreatic cancer liver metastasis [67]. The authors showed that the neutrophils that infiltrated the liver metastatic lesion were P2RX1 negative, while in the normal liver tissue, the neutrophils expressed P2RX1, an ATP receptor that participates in metabolism and energy generation. From the perspective of neutrophils, P2RX1^−^ neutrophils expressed higher levels of the PD-L1 and ARG1, which is considered a potential mechanism for the immunosuppressive microenvironment in PDAC. Additionally, considering the similarity between PMN-MDSCs and neutrophils in terms of surface markers, previous studies on PDAC neutrophils merit careful and repeated verification to determine whether the conclusions are reliable. This evidence underscores the urgent need for researchers to analyze neutrophils with finer resolution, aiming for more precise interventions with neutrophil-targeted immunotherapeutic strategies.

### Targeting these signals, neutrophils have been decoded at single-cell resolution

Bianchi et al. were among the first to study the communication between neutrophils and cancer cells in the PDAC TME at the single-cell transcriptomic level, considering neutrophils as early sentinels in PDAC detection. They revealed a “cell-autonomous” interaction of CXCL1 with CXCR2^+^ neutrophils, and silencing CXCL1 reprogrammed the trafficking and functional dynamics of neutrophils to overcome T cell exclusion and control tumor growth in a T cell-dependent manner. The authors argued that TNF originating from neutrophils is a core factor in immune reprogramming and that the TNF-TNFR2 interaction causes excessive CXCL1 production, T cell dysfunction, iCAF polarization, and other microenvironmental changes, inducing PDAC immune tolerance and chemotherapy resistance [68]. This work suggests possible mechanisms of interactions between neutrophils and other cells in the PDAC TME, and although interventions targeting secretory signals such as TNF in the TME often lack specificity due to their broad impacts, this research still provides evidence for the pivotal role and therapeutic potential of targeting neutrophils in the TME. A novel mechanism by which the EHF-CXCL1/CXCR2 axis is regulated by nifurtimox to suppress the recruitment of MDSCs to the PDAC TME was proposed. Moreover, nifurtimox can also inhibit the JAK/STAT pathway to further inhibit inflammation and suppress CXCR2^+^ neutrophils, ultimately improving resistance to chemotherapy and immunotherapy [69].

Despite numerous challenges in the single-cell transcriptomics of neutrophils, researchers have conducted extensive sequencing and analyses of neutrophils in solid tumors, revealing their significant role in the liver cancer immune microenvironment [59]. However, similar mapping and typing efforts in PDAC are still in their initial stages. Wang et al. revealed the heterogeneity between PDAC neutrophils and peripheral circulating neutrophils at the single-cell level and to classify PDAC neutrophils. Tumor-associated neutrophils (TANs) were divided into five groups based on their marker genes (Table 2): PMN (circulating neutrophils), TAN-3 (cells transitioning from the periphery to the tumor), TAN-0 (functionally unspecified), TAN-4 (preferentially express interferon-stimulated genes), TAN-2 (inflammatory), and TAN-1 (terminally differentiated with protumor functions, highly glycolytic, and regulated by the BHLHE40 transcription factor). This classification model seems to reconcile traditional views on the protumorigenic and antitumorigenic roles of neutrophils. Through the regulation of the BHLHE40 transcription factor and stimulation by the hypoxic transcription factor HIF1A under hypoxic conditions, TAN-1 activates downstream genes such as LDHA, PLAU, and VEGFA, promoting a local glycolytic and hypoxic microenvironment that leads to immune suppression. Evidence also suggests that BHLHE40 activates the expression of IL1RN and PDE4B in TAN-2, which may regulate the differentiation of TANs within the PDAC TME. The level of TAN-1 infiltration correlates with poor patient outcomes, while other TANs functions may be linked to tumor cell killing, potentially playing a role in inhibiting tumor growth and thus providing deeper insights into the longstanding debate over neutrophil infiltration and the PDAC patient prognosis.

Notably, in this study, the neutrophils were sorted as CD45^+^CD66b^+^ before they were subjected to single-cell sequencing, which may differ from the results obtained for neutrophils annotated after direct sequencing of the complete sample. Furthermore, the differentiation trajectories in this study are data-driven and cannot be confirmed in vivo regarding TANs development and differentiation pathways. Melissa and colleagues addressed this issue using a PDAC mouse model and scRNA-seq technology in their latest research, proposing a new classification of tumor cells [12]. Before classifying neutrophils in the PDAC TME, neutrophils derived from outside the TME, including the bone marrow, peripheral blood, and spleen, were shown to be significantly different from those within the TME, and TME neutrophils developed from preNeu cells and immature neutrophils. Neutrophils in the TME were categorized into three groups based on cell surface markers: T1 (CD101^−^, dcTRAIL-R1^−^), T2 (CD101^+^, dcTRAIL-R1^−^), and T3 (dcTRAIL-R1, regardless of CD101 expression). These groups also exhibited the following transcriptional characteristics: T1 highly expressed the genes Ltc4s, Mmp8/9, Ppia, Prr13, Ptma, and Retnlg, which are associated with rRNA processing, proton transmembrane transport, and nucleoside diphosphate phosphorylation; T2 highly expressed genes such as Cd300ld, Cxcr2, Dusp1, Gbp2, Ifitm1, Il1b, Isg15, Jaml, Junb, Msrb1, Osm, S100a6, Selplg, and Slpi, which are linked to ROS metabolic regulation, amino acid metabolism, the immune response, cell proliferation, and transcriptional regulation; and T3 highly expressed Atf3, Ccl3, Ccl4, Cd274, Cstb, Cxcl3, Hcar2, Hilpda, Hk2, Hmox1, Ier3, Jun, Plin2, Spp1, Tgif1, Tnfrsf23, Vegfa, Zev2, Ldha, and Mif, which are associated with oxidative stress, hypoxia, glycolysis, angiogenesis regulation, protein folding, and translation.

Functionally, compared to the previous classification by Wang et al., the core T3 neutrophils resemble the TAN-1 subtype, with strong BHLHE40 transcriptional activity and the expression of genes related to hypoxia, glycolysis, and angiogenesis, providing further evidence of the existence of terminally differentiated neutrophil subtypes in the PDAC TME. T2 neutrophils might resemble the inflammation-related cells proposed by Wang et al., while T1 cells could represent an intermediate state of proliferation and migration. Except for maturity, neutrophils entering the PDAC TME can differentiate into T3 cells, and this differentiation is unidirectional and irreversible, suggesting that all neutrophils migrating into the TME ultimately differentiate into T3 cells and maintain this phenotype to promote tumor growth. This finding might explain why, despite the high heterogeneity of neutrophil infiltration in the PDAC TME, neutrophil infiltration is generally associated with a poor prognosis; hence, targeting T3 differentiation, such as using anti-dcTRAIL-R1 monoclonal antibodies, may be pivotal. The biological characteristics of T3 cells are also noteworthy. T3 cells are primarily located in areas within tumors with high hypoxia and glycolytic activity, where VEGF*α* expression is upregulated, inducing angiogenic remodeling in the necrotic core of the tumor. Interestingly, the half-life of T3 neutrophils is significantly longer than that of other neutrophils, and the expression of the cell surface protein marker dcTRAIL-R1 gradually increases after these cells migrate to the periphery and can be maintained for at least five days, which contradicts the traditional view of the very short lifetime of neutrophils, indicating that T3 neutrophils are uniquely influenced by the PDAC TME.

### Different sequencing equipment and sampling strategies might cause biased neutrophil capture

Interestingly, previous studies have shown that 10x technology has a low capture rate for neutrophils, while the BD Rhapsody single-cell sequencing platform could compensate for this shortcoming [70]. However, recent studies on neutrophils in PDAC have employed cell surface marker enrichment followed by sequencing on the 10x platform [12], while another study in which a similar protocol was applied did not identify any neutrophils [56]. Whether methodological differences and preferences could bias research and classification methods for neutrophils in pancreatic cancer remain unclear. Given the short lifespan and high plasticity of neutrophils, the impact of post sorting sequencing on certain neutrophil subgroups is also uncertain. We are now able to initially delineate the landscape of TANs in PDAC at the single-cell transcriptome level and have begun to understand the migration and differentiation patterns of TANs in PDAC, suggesting that the development of precise neutrophil-based targeted therapies for PDAC is on the horizon.

**Table 2:** The markers and functions of neutrophil subclusters identified in the PDAC TME

Neutrophil Types	Markers	Functions
Pan-Neutrophil [10, 12, 71]	CD66b, FCGR3B	
TAN-0 [10]	Not Available	No distinctive features
TAN-1 [10]	VEGFA, PLAU, LGALS3, BHLHE40, LDHA	Pro-tumor function; terminally differentiated; high glycolytic activity
TAN-2 [10]	NLPR3, CD69, PDE4B, IL1RN, ADM	Inflammation associated
TAN-3 [10]	S100A8, S100A9, MME, VMN2, SELL	Transitional stage
TAN-4 [10]	IFIT1, IFIT2, IFIT3, ISG15, RSAD2	Preferentially expressing interferon-stimulated genes
T1 [12]	Ltc4s, Mmp8, Mmp9, Ppia, Prr13, Ptma, Retnlg	rRNA processing; Proton transmembrane transport; nucleoside disphophate phosphorylation
T2 [12]	Cd300ld, Cxcr2, Dusp1, Gbp2, Ifitm1, Il1b, Isg15, Jaml, Junb, Msrb1, Osm, S100a6, Selplg, Slpi	Regulation of process of cellular amide, ROS metabolic, cell proliferation and translation by RNA polymerase II (negative); Immune response
T3 [12]	Atf3, Ccl3, Ccl4, Cd274, Cstb, Cxcl3, Hcar2, Hilpda, Hk2, Hmox1, Ier3, Jun, Plin2, Spp1, Tgif1, Tnfrsf23, Vegfa, Zev2, Ldha, Mif	Response to oxidative stress and hypoxia; Positive regulation of angiogenesis; Glycolytic process; Protein folding and Translation

## Mast cells participate in TME remodeling and response to chemotherapy

Mast cells, which are rich in histamine and heparin granules, release a multitude of cytokines, leukotrienes, and proteases into the TME, where they exert bidirectional regulatory effects on immune responses [72]. Descriptions of mast cells in the PDAC TME are sparse, with few reports on their local specific functions within the TME. Surprisingly, many scRNA-seq studies of PDAC have noted the presence of mast cell subpopulations [9, 38]. The proportion of mast cells in the TME remains unclear; while early studies suggested a significant increase in mast cells infiltration in PDAC tissues compared to that in peritumoral tissues [73], recent findings indicate a significant decrease in mast cells within tumor regions [56], although these studies were not conducted at the single-cell level.

Early researches on cell lines analyzed the role of mast cells in the TME, indicating that they promote the growth of pancreatic stellate cells (PSCs)and tumor cells by secreting IL-13 and tryptase and facilitate tumor cell metastasis in an MMP-dependent manner; conversely, mast cells are also activated and migrate upon stimulation by tumor cells [74]. Recent studies have suggested that in the TME, mast cells marked by KIT and CPA3 can induce the release of TGF-β, activate Smad4 signal transduction, upregulate PAR-2 and ERK1/2 expression, and activate AKT signaling, thereby inhibiting tumor cell apoptosis and inducing resistance to gemcitabine [10, 19, 38, 75]. Some studies also suggest that mast cells activation are associated with angiogenesis in PDAC, although only correlations have been reported [76]. Although targeting mast cells migration and function in mouse models can suppress PDAC growth, direct evidence of the effects of mast cells on the TME is lacking.

As increasing attention is directed toward inflammatory signaling and various cytokines in the TME and their impacts on PDAC, the response of mast cells to cellular signals and their secretory capacity within the TME merit further investigation. Moreover, mast cells might contribute to the immunosuppressive microenvironment by regulating components of the PDAC matrix, such as proteases, by modulating PSCs proliferation, and potentially by regulating CAFs, making the alteration of extracellular matrix (ECM) components involving mast cells a potential strategy for precise intervention in PDAC.

## Dendritic cell-classified neoantigens

At the end of the myeloid phase, we discuss dendritic cells (DCs), which are the principal antigen-presenting cells in the immune system and play a crucial role in bridging innate and adaptive immunity. Notably, DCs play a crucial role in PDAC. They can recognize tumor-specific peptides on the tumor surface. After selection, they can be reinjected into the body to effectively promote the generation of antigen-specific type I T cells, thereby enabling the organism to recognize and attack tumor cells [77]. Professor Steinman, a renowned patient with PDAC lymph node metastasis, personally tested this technique, extending his survival by more than four years. This approach has also become one of the early models for popular tumor vaccines and CAR-T cell therapies.

Unfortunately, subsequent research has shown that although DCs can recognize tumor cells and activate adaptive immune responses in many cancers, DCs are scarce and of lower quality in PDAC, making the activation of effective adaptive tumor immune therapies more difficult and resulting in the formation of an immunosuppressive microenvironment. The expression of PDAC neoantigens also promotes PDAC progression and metastasis through mechanisms such as increased collagen deposition via IL-17 signaling, activation of inflammatory pathways, EGFR ligands, and increased levels of granulocyte chemotactic factors [78]. The use of scRNA-seq technology to identify CD45^+^ immune cells revealed the subtypes and proportions of DCs in PDAC, identifying cDC1-type DCs that express high levels of CCL17 and IL8 but low levels of antigen cross-presentation genes such as HLA-A, HLA-C, TAP2, and PSMB9 as the primary type responsible for presenting antigens to CD8^+^ T cells, possibly promoting the migration of regulatory T cells and angiogenesis. In contrast, cDC2s are the main infiltrating DCs in the PDAC TME and express fewer costimulatory molecules and cell maturation markers, leading to a weaker antigen-presentation capacity. This phenomenon has been reported for cDC3-type DCs; however, these cells resemble an unidentified DC cluster [38]. This diversity may be one of the reasons why T cells in the PDAC TME are unable to receive neoantigens presented by DCs and fail to initiate cytotoxic responses. Another reason is that cDC1s in PDAC express relatively lower levels of CXCL9 than those in other malignancies, which weakens the recruitment of CD8^+^ T cells mediated by CXCL9-CXCR3 signaling. In addition to cDC1/2s, three other DC subtypes have been identified: MoDCs, pDCs, and mregDCs [56] (Table 3).

However, interventions targeting the infiltration and antigen-presenting capabilities of DCs to impact PDAC tumor growth have multiple potential applications. DCs are spatially located at tumor margins [56], and after depleting DCs, CD4^+^ T cells in PDAC differentiate into Th17 cells, which secrete more IL-17 and promote tumor growth and metastasis. A viable, improved method is to use Flt3L to increase the number of cDCs, which has shown potential in reversing the tumor matrix changes and tumor progression caused by neoantigens in early-stage PDAC; however, in advanced PDAC, the use of Flt3L alone is insufficient and needs to be combined with a CD40 antibody and radiotherapy to enhance cDC function and fully release neoantigens via a tri-therapy approach, but this method is feasible only in mice and lacks further research [78]. Another possible mechanism is that Smad4 deficiency can enhance the immunogenicity of tumor cells, significantly increasing the activation level of DCs, and this activation of DCs is not affected by the expression of tumor cell antigen-presenting genes, such as β2M [79]. Additionally, the use of tumor cell membrane-coated responsive nanogels can induce the recruitment and maturation of DCs through a mechanism dependent on activated NK cells, ultimately stimulating the activation of CD8^+^ T cells for antigen-specific tumor cell killing combined with NK cell-mediated nonspecific tumor cell killing to comprehensively suppress tumor growth [80].

**Table 3:** The markers and functions of dendritic cell subclusters identified in the PDAC TME

DCs Types	Markers	Functions
Pan-DC [56]	HLA-DRA, MRC1	
cDC1 [38, 56]	CLEC9A, XCR1	Antigen presentation
cDC2 [56]	CX3CR1, CD14, LGALS3, TGFB2	Primary DCs in PDAC TME
MoDC [56]	CD1C, CD1A, LGALS8, TNF, IL23A, HLA-DQB2, HLA-DPB2	High expression of pro-inflammatory cytokines
mregDC [56]	LAMP3, CD40, CD80, CD83, CCR7, CD274, PDCD1LG2, STAT3, IL6, IDO1, LGALS9	Associated with DC maturation and immunoregulation
pDC [38, 56]	CLEC4C, IL3RA, IRF7, ICOSLG, TGFB1, LILRA4, PLD4	plasmacytoid DCs, secreting interferon

## Lymphocytes exert direct tumor-killing effects

As previously mentioned, in addition to endogenous cell death pathways [81], almost all tumor-targeted therapeutic strategies ultimately activate one of the body’s most powerful immune killing mechanisms: the activation of CD8^+^ T cell cytotoxicity [82, 83]. Infiltrated CD8^+^ T cells are believed to be highly exhausted in the PDAC TME and clearly form an immunosuppressive TME [84]. A recent study documented that the combination of KRAS inhibition and an ICB strategy could effectively control PDAC progression and increase the infiltration of active T cells, especially in advanced PDAC [48]. Several immunotherapy strategies have been developed based on this concept [84–86]. Therefore, we will not further elaborate on how various cell components activate or suppress various subtypes of T cells to achieve their envisioned strategies for controlling tumor growth and metastasis. However, B cells, which are components of adaptive immunity that are as important as T cells, have received far less attention in PDAC than T cells.

B cells play a pivotal role in adaptive immune regulation, and evidence of B cell-related autoimmune responses in tumors has been published. However, B cell responses do not seem to affect tumor progression [87]. B cells can produce autoantibodies against tumor-associated antigens (TAAs), which may precede the symptoms of the disease and persist for months or years. PDAC plasma and PDAC cell-derived exosomes can significantly trigger such autoantibody responses, exerting a B cell-dependent decoy function against complement-mediated cytotoxicity. Conversely, exosomes can also promote the expansion of immunosuppressive B cell subpopulations [87]. A spatial analysis of B cells in the PDAC TME revealed that the arrangement of B cells was scattered but reduced near malignant ductal cells, which might decrease the generation of antibodies against TAAs and induce immunosuppression.

In studies applying scRNA-seq to the PDAC TME, unlike the higher capture rates of mast cells, many reports do not mention the presence of B cells and plasma cell subgroups. Furthermore, in studies that do report B cells and/or plasma cells, their proportions are relatively low and vary greatly between different samples and studies (5.75% [9]− 8.93% [88]). Initially, Peng et al. defined B cell subgroups in the PDAC TME based on the expression of MS4A1 [9] but did not detail their functions. Subsequent studies characterizing B cell infiltration and classification in the PDAC TME have produced many contradictory findings, likely related to patient heterogeneity and tumor progression. Another study sampled patients with different TNM stages of PDAC and revealed no B cell infiltration in early-stage PDAC (TNM stage I) but gradual infiltration into the TME and progression into subgroups with distinct gene expression profiles and pro- or anti-tumor functions. They noted the absence of regulatory B cells (traditionally marked by CD1D, CD5, and TGFBI) in the TEM, instead finding a predominance of plasma cells and memory B cells with high CD27 expression [88]. Moreover, B cells are also marked with MS4A1 and CD20 and are classified as naive B cells, memory B cells, or plasma cells, with both naive and memory B cells expressing TGFBI at high levels [56]. These controversies urgently require further single-cell studies to clarify and validate the role of B cells in the PDAC TME.

Additionally, although some studies using scRNA-seq have reported that B cell infiltration is correlated with the expression of biomarkers such as GFPT2 and GPRC5A or is associated with a poor prognosis for PDAC patients, the analyses of these data often rely on immune infiltration assessment algorithms such as TIMER; thus, these conclusions are simplistic associative analyses and somewhat unreliable [89, 90]. Convincingly, PDAC patients with greater B cell infiltration had significantly shorter survival periods, and in a KC mouse model, IL-1β was found to induce PD-L1 expression in B cells, increasing the proportion of regulatory B cells, forming an immunosuppressive microenvironment, and promoting tumorigenesis. Another study revealed that depleting B cells with a KRAS small-molecule inhibitor did not significantly improve tumor progression, suggesting that B cell-associated PDAC progression occurs in a KRAS mutation-independent manner [48]. Moreover, spatial resolution studies have shown that B cells are uniformly distributed in the TME but less so near the tumor epithelium; B cells expressing high TGFBI levels, which are similar to regulatory B cells, interact more closely with CD8^+^ T cells and macrophages [56], suggesting that B cells regulate tumor progression in the PDAC TME through cytokine and chemical stimulation rather than KRAS-related proliferation signals.

## Decoding the stromal component in the TME: restricting tumor cells

As initially mentioned, almost all therapeutic strategies act directly or indirectly on ductal epithelial cells; thus, we do not focus solely on ductal cells here. An early analysis categorized ductal cells into two types based on cell markers and copy number variations: ductal cell 1, marked by AMBP, CFTR, and MMP7, and ductal cell 2, marked by KRT19, KRT7, and SLPI, with the latter identified as malignant epithelial cells in tumors [9]. With the deepening of studies based on scRNA-seq, an increasing number of markers have been identified on ductal cells that can predict clinical indices such as immunotherapy sensitivity and survival in PDAC patients [16, 37]. By performing a spatial transcriptomics analysis, the spatial heterogeneity of ductal cells was also profiled. Most ductal cells were localized in normal duct regions, while hypoxic ductal cells and malignant ductal cells were enriched in tumor regions and might be educated by signals from the surrounding TME [41]. Moreover, with the use of organoid models, which are primarily composed of epithelial cells [17], numerous phenotypic studies targeting malignant epithelial cells in PDAC have been conducted, especially those using organoid platforms for rapid, high-throughput drug screening [91]. Another patient-derived xenograft model was also used to evaluate the effect of KRAS inhibition, and growth inhibition was observed after treatment [48]. However, TGF-β signaling was upregulated after treatment, and the addition of TGFβ1 resulted in resistance to MEK inhibitors [92]. The authors also confirmed that TGF-β signaling contributed to the bypass of the KRAS pathway by promoting the Epithelial-Mesenchymal transition, and the activation of RTK-PI3K-AKT signaling was another potential pathway for promoting tumor cell growth. The EMT and angiogenesis pathways were also enriched in tumor cells after chemotherapy, which jointly suggested potential mechanisms for the recurrence and progression of tumors [38].

**Table 4:** The markers and functions of fibroblast subclusters identified in the PDAC TME

Fibroblast Types	Markers	Functions
Pan-CAF [38, 71]	COL1A1, FAP, PDPN, DCN, VIM	
myCAF [71, 93]	ACTA2, TAGLN, MMP11, MYL9, HOPX, POSTN, TPM1, TPM2	Collagen deposition
iCAF [71, 93]	IL6, PDGFRA, CXCL12, CFD, DPT, LMNA, AGTR1, HAS1, CXCL1, CXCL2, IL8	Inflammation related
apCAF [71]	CD74, HLA-DRA, HLA-DPA1, HLA-DQA1, SLPI	Antigen-presenting
Mucciolo et al. [94]	myCAF markers but CD90 negative	Pro-metastasis, affected by EGFR/ERBB2 signal
Niu et al [62]	SETD2 deficiency, ABCA8a	Adipogenesis, Spatial proximity to tumor cell
CD133^+^ iCAF [95]	PROM1(CD133), MET, EPCAM, CD24, CD44	Related to chemotherapy
CXCR4^+^ iCAF [95]	CXCR4, CD44	Related to chemotherapy

While extensive research on metabolomics, proteomics, and epigenomic regulation also exists beyond scRNA-seq, focusing on tumor cell lines and organoid models, these studies, although not discussed here, greatly aid in understanding the biological functions of various components of PDAC at the single-cell level, including ductal cells [95–97]. Next, we discuss acinar cells, which are located in the pancreatic ducts alongside ductal cells and play a key role in the PDAC TME; however, these cells have received much less attention.

### Acinar–ductal abnormal metaplasia leads to neoplasms

Since the widespread application of single-cell technologies, the canonical markers of acinar cells are PRSS2 and CTRB1 [9, 16]. Acinar cells have also been traditionally classified as i-Acinar, s-Acinar, and REG-Acinar (marked by REG3A and REG1B, related to pancreatic inflammation) [98]. However, these cells are rarely defined as distinct subgroups within the PDAC TME and appear only in conventional cell atlases. The function and proliferation of acinar cells in the PDAC TME differ markedly from those in the physiological state.

Under physiological conditions, ductal and acinar cells primarily arise from self-replication. However, under pathological conditions, a conversion between ductal and acinar cells can occur, which is termed acinar to ductal metaplasia (ADM) [99]. Endocrine cells derived from ADM are diverse and share similar marker genes in the pancreas and stomach [100]. ADM subgroups expressing HNF1B or POU2F3 may have greater potential for neoplastic transformation and can form MUC5AC^+^ gastric-pit-like cells. The presence of heterogeneous ADM leads to varying outcomes for acinar cells. Studies based on scRNA-seq have shown that tumor cells and acinar cells mutually and exclusively express ductal and acinar marker genes. ADM cells express a combination of marker genes for both cell types and may continue to differentiate into normal ductal cells and tumor cells. Researchers have attempted to analyze the results jointly using snRNA-seq and spatial RNA-seq, yet no definitive conclusions have been reached [101]. Additionally, currently, no evidence is available to suggest that the differentiation process of ADM is reversible.

In ex vivo acinar cell models [100], TGF-α can induce the transformation of acinar cells into ductal cells, forming duct-like tubular structures through induction via the insulin receptor (INSR). Increasing insulin concentrations significantly enhances this induction effect, while a protease inhibitor completely blocks the synergistic effect of insulin and TGF-α. The authors also confirmed that in acinar cells with a KRAS mutation [12], INSR is essential under conditions of high-fat diet-induced obesity for the hyperinsulinemia-driven formation of PanINs; INSR knockout can inhibit PDAC development associated with hyperinsulinemia [102]. Moreover, an exciting result showed that treating Kras^G12D^ mice with a Kras inhibitor was a promising approach for restoring PDAC tumors to normal pancreatic tissue [48]. Targeting the insulin receptor signaling pathway in acinar cell components may be an effective strategy for treating and preventing pancreatic cancer.

Single nuclear chromatin accessibility testing revealed that acinar cells are rich in transcription factors such as T cell factor/lymphoid enhancer factor 3/12 (TCF3/TCF12) and nuclear receptor subfamily 5 group A member 2 (NR5A2), which are likely related to the development and maintenance of pancreatic exocrine function [40]. The authors also found increased activity of the TEAD in cells undergoing ADM compared to conventional acinar cells, with TEAD transcription factors driving SRY-Box transcription factor 9 (SOX9, the transcription factor for ADM). These authors also provided functional annotations for several representative markers on the surface of acinar cells: GFI1 is related to acinar cell development, and FGF9 and FGF10 are involved in pancreatic development and are crucial for neuronal development and maturation. In the subgroups of cells undergoing ADM, distinct differences in marker gene expression have been detected, for example, PCDH1, which is involved in homophilic cell adhesion via the plasma membrane and is also a poor prognostic marker for PDAC [103], and HEG1, which encodes a membrane protein involved in cell‒cell junction organization and is a viable target in mesothelioma [40].

Although these markers may lack cell-type specificity, in the scRNA-seq results, they serve as annotations for biological functions and provide crucial clues about how these cells, at the “edge of investigation”, participate in the composition of the PDAC TME. This information is very important for studying the function of acinar cells at the single-cell level.

### Fibroblasts induced bidirectionally with plastic polarization and migration

Fibroblasts are among the most prevalent stromal components within the PDAC TME, and, correspondingly, PDAC tumors are often characterized by a dense and fibrous environment, which differs from that of other tumors that are rich in newly formed blood vessels and tumor cells. Many studies have investigated interventions for PDAC progression through the depletion of cancer-associated fibroblasts (CAFs), yet these studies conducted at bulk resolution have yielded many inconsistent conclusions [104].

With continuous advancements in single-cell sequencing technologies, research on CAFs in PDAC has revealed various classifications of CAFs subtypes. Tuveson et al. reported that CAFs can generally be categorized into three types: myCAFs, which primarily express αSMA; iCAFs, which exhibit a high secretory capacity; and apCAFs, which have been shown to have antigen-presenting capabilities [71, 93]. In research related to single-cell transcriptomics, defining subpopulations solely based on single-cell markers is often inaccurate. Notably, αSMA (ACTN2), which is expressed by myCAFs, is also widely expressed in other cells [101]. Although some studies propose specific markers and novel classification methods, these classifications are mostly dataset-dependent and are not universally applicable to general research datasets [88, 105]. These classification methods might also overlap with those proposed by Tuveson. Although numerous studies have proposed various classifications, their categorization into myCAFs, apCAFs, and iCAFs is widely accepted by most scholars. In summary, CAFs, as highly heterogeneous components, play multifaceted and often controversial roles in tumor development and progression.

One of the primary sources of myCAFs is PSCs. In the PDAC microenvironment, PSCs can be activated by tumor cells and immune cells, leading to the disappearance of vitamin A lipid droplets in the cytoplasm and the upregulation of αSMA expression. When cultured in vitro, these cells typically acquire a myCAF phenotype [106]. PSCs are the main cells producing type I collagen in the ECM in PDAC. A reduction in the collagen content can accelerate the development of PanIN and PDAC and decrease overall survival, which is associated with the upregulation of CXCL5 expression in tumor cells due to the absence of type I collagen through a mechanism mediated by SOX9 and may ultimately lead to the recruitment of MDSCs and the suppression of CD8^+^ T cells [67]. Therefore, targeting the transition between PSCs and CAFs can also regulate the progression of PDAC through one key mechanism, the Rho effector protein kinase N2 (PKN2) pathway, which will be discussed later.

Generally, myCAFs tend to suppress tumor growth, while iCAFs tend to promote tumor growth. These differences lead to the following questions: Could the phenotype of CAFs undergo transformation? Could such transformations aid in controlling tumor growth? CAFs phenotypes can interconvert under different stimuli and culture conditions, posing significant challenges for long-term studies of CAFs isolated from patient tissues [71, 93]. Early research indicated that iCAFs gradually transition to a myCAF phenotype and express myCAF-associated genes when cultured in a monolayer. Similarly, TGFβ stimulation can also mitigate the inflammatory effects of the JAK/STAT pathway, weakening the iCAF phenotype and shifting it toward myCAFs.

Recent single-cell studies have significantly identified the mechanisms underlying the plasticity of such CAFs phenotype transitions. The relationship between TGF-β and IL-1 in the CAFs phenotypic transformation has been reported in both organoid and mouse models [19, 107]. Specifically, IL-1 induces LIF expression and downstream activation of the JAK/STAT pathway to induce iCAF production, while TGFβ counteracts IL-1 stimulation by downregulating IL-1R expression, inhibiting the iCAF phenotype and promoting differentiation into myCAFs. Notably, IL-1 is secreted through multiple pathways, and many researchers have focused on IL-1 secreted by tumor-associated macrophages and malignant ductal epithelial cells in PDAC [19]. Targeting IL-1 may constitute a promising multitarget intervention approach.

TGF-β plays a significant role in myCAFs [94]. EGFR/ERBB2 was introduced in this study, and therapies targeting EGFR (such as erlotinib, an FDA-approved drug used for advanced pancreatic cancer) are among the most effective treatments for PDAC [108, 109]. Researchers have shown that TGF-β in myCAFs can activate EGFR/ERBB2 signaling via an amphiregulin-mediated autocrine process, providing new theoretical support for EGFR-targeted therapy. Moreover, the only ligand involved in the ERBB2 pathway is neuregulin-1 (NRG1), and the expression of NRG1 on CAFs was shown to result in resistance to KRAS inhibitors [110]. Researchers also reported the upregulation of ERBB2 and ERBB3 in PDAC samples after KRAS inhibition, and most patients with recurrent tumors were examined for novel KRAS or downstream RTK/PI3K/MAPK pathway mutations. Another group used PDAC organoids and mouse models to study the impact of EGFR signaling activation on different CAFs subgroups and discovered that inhibiting EGFR/ERBB2 signaling does not affect all myCAFs but rather specifically affects those that are CD90^+^.^108,109^ Interestingly, the activation of myCAFs by EGFR can promote the metastasis of mouse PDAC, in contrast to the traditional view that myCAFs inhibit cancer progression, indicating that significant heterogeneity still persists within the current CAFs classifications.

Evidence of transitions from myCAFs to iCAFs has also been documented, with many research findings focused on PSCs [111]. These findings suggested that the transition from myCAFs to iCAFs may be due to changes in the ratio of PSCs that differentiated into different CAFs subtypes. An analysis of specific CAFs subtypes resulting from PSCs-to-CAFs transformation indicated that both in vivo and in vitro, the absence of PKN2 inhibited PSCs proliferation, reduced αSMA expression, induced a transition from myCAFs to iCAFs and prevented PSCs invasion but promoted tumor growth when cocultured with PDAC tumor cells [111]. Moreover, IL-17A^+^ CD8^+^ T cells secrete IL-17A, which, in conjunction with TNF signaling, can induce iCAFs and promote tumor progression [112]. In vitro validation with cell lines revealed that coculture of Tc17 cells with quiescent PSCs upregulated the expression of CXCL1, IL6, LIF, SAA3, CSF3, and Ly6C, indicating that quiescent PSCs respond to inflammatory signals and that these cells can differentiate into iCAFs rather than myCAFs.

However, beyond the classic classifications of CAFs, researchers continue to propose new CAFs subtypes based on metabolic and epigenetic data. They hope to study CAFs at spatial resolution, but the size of the basic analysis unit “spot” of the widely used spatial transcriptomics technologies has not reached the single-cell level. Current studies mainly analyze cell infiltration at spatial resolution by analyzing predominant but not specific markers through spots. For example, studies have shown that CAFs marked with SFRP1 are distributed in the periphery of PDAC tumors, while those marked with LRRC15 are located mainly in the core areas of the tumor [101]. However, these conclusions cannot be drawn at single-cell resolution.

For instance, through single-cell sequencing of mouse pancreatic tumors and experiments testing metabolic function, a study focused on the epigenetic regulation of CAFs revealed that pancreatic tumor cells with a KRAS mutation combined with SETD2 deficiency exhibit metabolism predominantly based on oxidative phosphorylation [62], in contrast to tumor cells with KRAS combined with P53 mutations, which primarily utilize glycolytic metabolism [113]. This group of CAFs displays characteristics that are markedly different from those of previously reported CAFs subtypes: enrichment of the adipogenesis pathway with a high content of neutral lipids, expression of the specific marker ABCA8a and spatial proximity to tumor cells with expression patterns suggesting strong BMP2-BMPR signaling communication. Another study reported that KRAS inhibition in mice increased the infiltration of CAFs and that the proportion of CAFs was similar to that in the normal pancreas, which could be regulated by immune cells infiltrating the PDAC TME [48]. However, resistance to KRAS inhibitors is also common. The effects of targeting KRAS signaling or downstream MEK signaling were temporary, but most tumors recurred soon after [110]. CAFs were shown to play a key role in promoting resistance to KRAS inhibitors. The resistance to chemotherapy is also mediated by CAFs. The interaction between iCAFs and TAMs, such as CXCL12-CXCR4 signaling, which are the most active ligand and receptor in the treatment of the naive PDAC TME, was significantly decreased [38].

We summarize both classic and novel CAFs markers used to define subtypes to better understand these classifications (Table 4). However, these subtypes often lack cross-matching with previous classifications, making ascertaining whether these findings represent entirely new subtypes or whether they belong to an existing subtype but express specific marker genes difficult. On the other hand, gene dropout is one of the main issues of single-cell techniques; therefore, some genes might not be captured in a few cells, which leads to mistakes when annotating cell clusters. Regulatory network- and gene set-based classifications could be applied to overcome the lack of marker genes, and this issue was improved by increasing the number of captured cells.

**Fig. 3:**
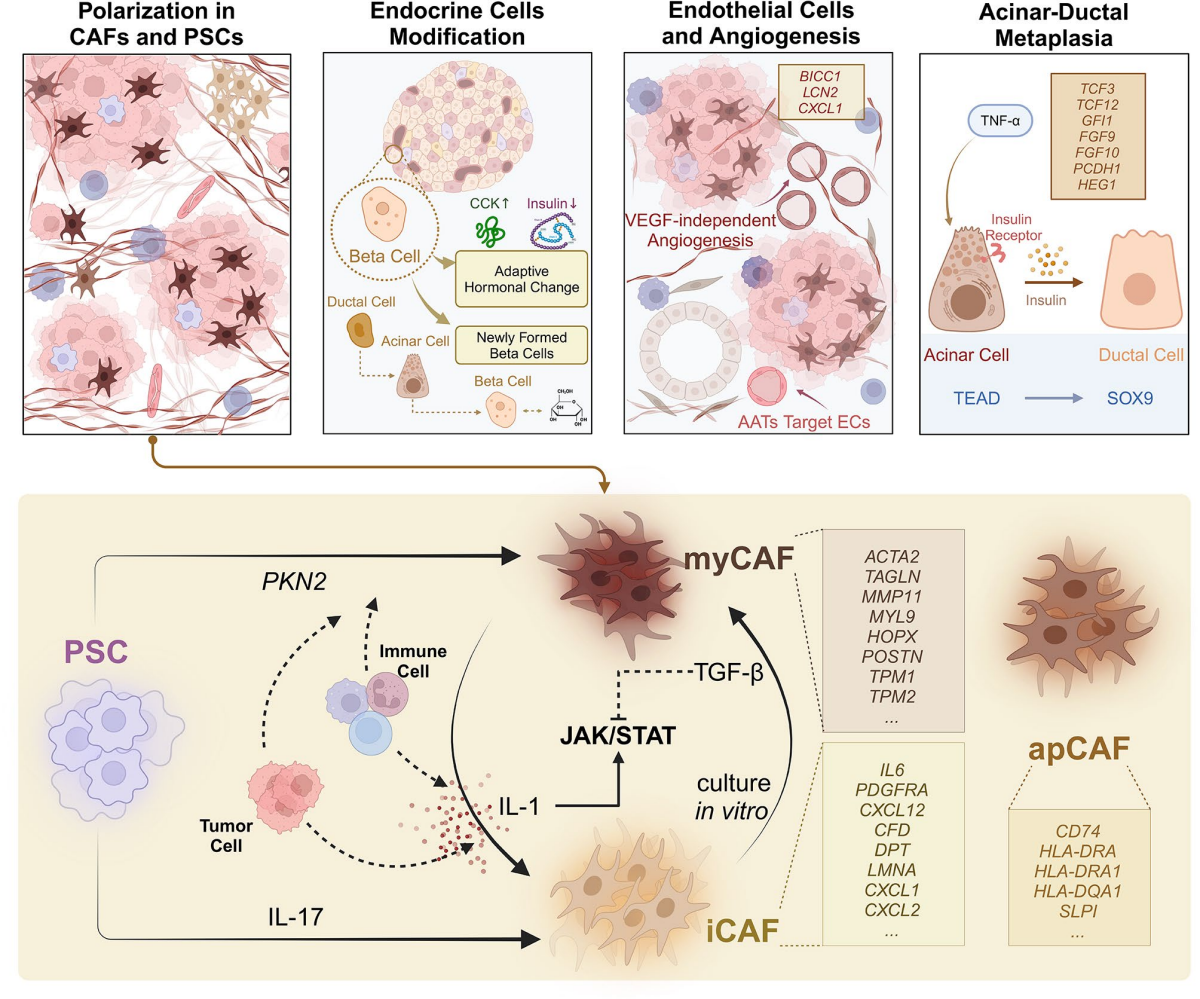
Landscape of stromal cells in the PDAC TME. Several biological processes related to tumorigenesis are depicted: polarization in CAFs and PSCs, endocrine cell modification, endothelial cell-mediated angiogenesis, and acinar-ductal metaplasia. The CAFs differentiation trajectories and key regulatory pathways are highlighted with canonical marker genes. The bidirectional phenotypic shift between myCAFs and iCAFs is driven by diverse cytokines and signaling in the PDAC TME. The markers for apCAFs are also displayed above

In summary, CAFs in the PDAC TME exhibit unique biological behaviors and classification standards. The classical categorization of CAFs may still be imprecise, with considerable heterogeneity existing within myCAFs and iCAFs. Different PSCs differentiate into myCAFs or iCAFs following stimulation with signals such as TGFβ or interleukins and migrate spatially to specific areas. This differentiation and migration process is plastic, and the cells can be remodeled by blocking cytokines or additive stimuli (Fig. 3). The current classifications of CAFs fail to adequately depict their responses to the TME and their contributions to the progression of PDAC; hence, this limitation leads to a significant issue: simply targeting a specific type of CAFs, such as depleting all myCAFs, is an inadequate treatment strategy that does not precisely control the progression of PDAC. Therefore, more research into the spatial and temporal heterogeneity of CAFs, the roles of different CAFs surface markers, and the regulatory mechanisms of CAFs activation or suppression signals at the single-cell level is urgently needed.

### Endocrine cells modified by metabolism and the nervous system

Research on endocrine cells (ECCs) within the PDAC TME is relatively scarce. This lack of research may be related to the location of PDAC onset and the sites selected for surgical sampling. Some studies have identified mature islet cells using gene markers such as GCG (alpha cells), INS, IAPP, and CHGA (beta cells) [9]. Endocrine disorders such as type 2 diabetes and obesity are among the high-risk factors for PDAC because they alter pancreatic endocrine function. Insulin signaling also plays a significant role in pancreatic cancer, with evidence suggesting that it contributes to PDAC progression by inducing obesity and interfering with diet [114, 115].

Recent studies, however, indicate that ECCs in the TME also exhibit adaptability and plasticity. Under obese conditions, ECCs undergo adaptive hormonal changes, abnormally expressing cholecystokinin (CCK) in β-cells and reducing insulin production, thereby promoting PDAC progression [115]. Another study reported the plasticity of ECCs at the single-cell level through long-term culture of human pancreatic slices and revealed that treatment with BMP (BMP-7) can induce pancreatic ductal cells to first transform into acinar cells (marked by the expression of the CFTR/SPP1/KRT19 genes, with downregulation of OLFM4 and upregulation of ID3 expression) and subsequently form ECCs, and the authors confirmed that β-cells with newly formed ECCs can respond to glucose [116]. Studies have shown a close relationship between ECCs, acinar cells, and the autonomic nervous system and have elucidated the neural functions of surface marker genes of ECCs. For example, PAX6 is associated with neuron projection morphogenesis, ISL1 is a key gene involved in the function of β-cells, and the integrin encoded by ITGB1 is crucial for ECC proliferation and neuronal cell migration [40].

Overall, current research on ECCs within the PDAC TME primarily focuses on how metabolic pathways, such as insulin signaling, influence the progression of PDAC. Neural modulation and hormonal stimulation may also play significant roles. However, designing targeted intervention strategies for PDAC that focus on ECCs requires further in-depth study.

### Fewer endothelial cells respond to antiangiogenic therapies, and more exhibit immunoregulation

Endothelial cells (ECs) are aligned in a monolayer on the inner surfaces of blood and lymphatic vessels. In addition to their inherent function in controlling substance exchange, ECs also participate in the regulation of hemodynamics, coagulation, angiogenesis, and inflammatory processes [117]. In quiescent tissues, ECs are classically classified into arterial, venous, capillary, and lymphatic types, which display heterogeneity across different tissues. Regardless of the organ where the tumor occurs, ECs in the TME play crucial roles in promoting cancer and metastasis [118], leading to the widespread use of antiangiogenic therapies (AATs) for treating various types of malignancies [119]. However, AATs primarily target tip or proliferating ECs, and studies of lung cancer using scRNA-seq have revealed that these ECs might constitute less than 10% of the population. Conversely, these ECs possess many yet-to-be-defined immunoregulatory functions, necessitating further single-cell level studies and in vivo research to understand the specific mechanisms by which AATs inhibit tumors. Regrettably, despite numerous scRNA-seq studies on ECs, a standard nomenclature or classification system for ECs in the TME has yet to be defined; most research relies on specific surface markers, many of which lack EC specificity and functional annotation [117].

In the pancreas, many scRNA-seq studies have reported that ECs in the TME are annotated with high expression of PLVAP, VWF [9, 16, 19], CLDN [9], and PECAM1 [19, 38], but reports on the proportion and function of these ECs vary greatly, likely due to differences in PDAC sampling and sequencing methodologies [120, 121]. Recently, single-cell studies of ECs from the PDAC TME were reviewed, and potential targets, such as PLVAP, IGFBP3, ICAM1, VCAM1, and SELE, and corresponding clinical studies were identified [117]. Other studies have linked angiogenesis with PDAC progression, noting that high BICC1 expression in human pancreatic cancer tissues is associated with an increased vascular density, tumor growth, and a poor prognosis [122]. Specifically, BICC1 upregulates LCN2 expression and activates the JAK2/STAT3 signaling pathway, leading to increased expression of the angiogenesis factor CXCL1, and this angiogenesis is VEGF independent, potentially explaining why some patients respond poorly to AATs and providing new insights into ECs heterogeneity.

However, as previously noted [117], research on ECs in the PDAC TME is still flawed, as monoclonal antibodies targeting these markers cannot guarantee the specific recognition of ECs but can block the corresponding signals throughout the microenvironment, and thus defining the true role of ECs in the TME is difficult. ECs in PDAC primarily upregulate processes such as ECM remodeling, angiogenesis, and hypoxia responses, and these cells may undergo a reactive endothelial-to-mesenchymal transition. Despite being described as a cohesive subgroup, the internal heterogeneity of ECs in PDAC has rarely been analyzed. A possible approach is to compare changes in ECs subgroups in PDAC patients before and after AAT treatment to identify potentially AAT-sensitive subgroups. However, another challenge arises because the PDAC TME is hypovascular, meaning that ECs are relatively scarce, and capturing a sufficient number of ECs in small samples might be difficult, while increasing the sample size could lead to excessive heterogeneity among PDAC patients, a problem that also appears in other low-abundance subgroups.

### Promising integrated analysis strategies

#### Multimodal data integration

**Fig. 4:**
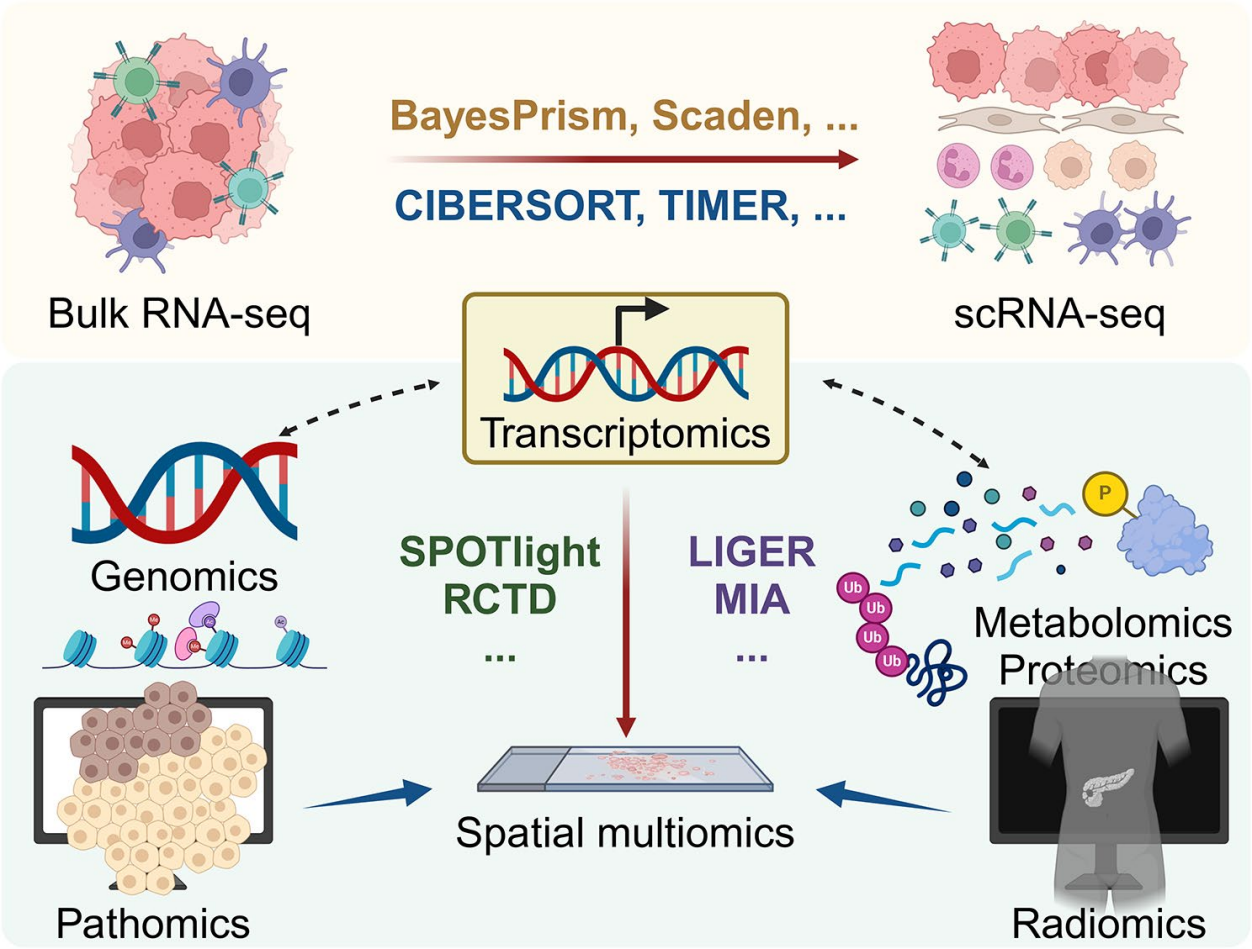
Integrated analysis strategies and models for multiomics

Research on the PDAC TME has entered the era of single-cell resolution with the rapid development of sequencing and mass spectrometry technologies. Here, we will discuss them from genomics, transcriptomics, and proteomics to other omics technologies (Fig. 4).

**Genomics-based integration.** First, genomics results are important but are often ignored. Initially, we proposed the application of genome and single-cell genomics to trace the origin of tumor cells, and novel sequencing methods, such as high-throughput chromosome conformation capture (HiC-seq), were proposed for profiling 3D chromatin maps and the lineage-specific regulatory architecture [123]. In addition, genomics results can be inferred from RNA-seq data by mapping the transcripts to the reference genome. Several tools have been developed based on this concept [124, 125].

**Transcriptomics-based integration.** Beyond genomics, integrated analyses of multiomics data such as scRNA-seq and scATAC-seq data are urgently needed. For instance, many studies utilize scRNA-seq to analyze the transcriptomic characteristics of tumor cells; however, most regulatory studies rely on algorithm-driven software packages such as SCENIC to identify potential targets for experimental validation [10]. rather than characterizing transcriptional regulatory potential using scATAC-seq. This approach largely depends on the completeness of reference databases provided by such software packages. In other tumor types, such as clear cell renal carcinoma, methods combining scRNA-seq with scATAC-seq have been used extensively to analyze regulatory characteristics across different tumor subtypes [14]. Our team recently showed that utilizing the integration of multiomics data, such as single-cell RNA-seq and bulk methylome, proteome and phosphoproteome data, from PDAC patients can effectively predict clinical outcomes and survival times, which suggested the promising clinical impact of this integration method [66]. However, in PDAC, limitations in sample collection and processing times may be reasons why such combined analyses have not yet been reported.

**Metabolomics and proteomics-based integration.** On the other hand, proteomics, the quantitative analysis of proteins that are the direct executors of most biological functions, is still in the methodological development stage at the single-cell level. Current methods based on single-cell Western blot analysis have low throughput and weak comparability of results, yet studies based on single-cell WB and scRNA-seq have shown unique advantages in cancers such as breast cancer [126]. An integrated model based on multiomics and lipidomics was also developed using machine learning methods and feature selection, but few studies have profiled the results at the single-cell level [127]. Traditional mass spectrometry-based proteomics still requires further development of microfluidics, chips, and other industrial technologies to achieve high-throughput single-cell resolution proteomic and metabolomic data. A new single-cell proteomics method using trapped ion mobility time-of-flight mass spectrometry was described [128], and the discovery application based on this platform reported that 3,140 total proteins were identified, with approximately 953 proteins quantified per cell, and a total of 1,498 single cells passed quality filtering [128, 129]. Although it has a relatively low degree of activity compared with that of approximately 20,000 genes and even many other proteins, evaluating inhibitors for specific KRAS mutations in PDAC is possible. Recent advances also include single-cell proteomics analysis methods developed with deep learning frameworks aimed at addressing batch effects, data noise, and missing data; however, their real-world performance still requires further validation [130]. By combining multimodal data, researchers have used multiplexed imaging and flow cytometry with ion exchange-based protein aggregation capture technology to characterize spatial proteomic heterogeneity with single-cell resolution [131]. They profiled the proteomic landscapes of 14 different cell types by sorting up to 1,000 cells from the same tumor. Other metabolites can also be detected using mass spectrometry at single-cell resolution. A rapid, label-free single‐cell analytical method based on active capillary dielectric barrier discharge ionization mass spectrometry was developed, which can analyze multiple metabolites in single cells at a rate of 38 cells per minute [132]. Using this method, abnormal lipid metabolism in pancreatic cancer cells was identified and verified at the mRNA level.

**Spatial omics-based integration**. Notably, most commercial spatial omics methods have not reached single-cell resolution to date, although several novel techniques are discussed here. The probe-based spatial sequencing method now enables spatial transcriptomic analysis at the subcellular level with a resolution of 2 μm to directly capture transcriptomic data, opening new doors for TME research. However to date, only a few studies have applied this method successfully [133]. A promising application is that most scRNA-seq analyses of cell–cell interactions are currently based on correlations of receptor–ligand expression in databases. However, due to the lack of spatial information in scRNA-seq data, many receptor–ligand pairs that require direct contact might only show correlational expression but are physically distant, leading to numerous false-positives. A novel package named novoSpaRc was designed to integrate spatial data and the expression patterns of ligands and receptors; however, its robustness should be further evaluated.

Single-cell RNA-seq is the most important and preferred solution for integration with spatial transcriptomics data. Using this approach, the lower depth of sequencing in the spatial transcriptomics data could be complemented by single-cell RNA-seq, and on the other hand, the lack of spatial information in the scRNA-seq data could also be added. However, integrating the scRNA-seq and spatial transcriptomics data is still challenging. Deconvolution (SPOTlight, RCTD) and mapping (LIGER, MIA and Seurat Integration) algorithms involving scRNA-seq and spatial RNA-seq data have been widely used to improve the resolution [41, 134, 135]. The PDAC TME was divided into three types by spatial transcriptomic and single-nucleus RNA-seq data according to the infiltrating cell type, and the relationships between the infiltration of immune cells and malignant cells were revealed [136].

Another method is to identify the cell boundary using staining and imaging methods, but the precise boundary must be determined by a deep learning-based identification algorithm [137, 138]. Radiomics and other image feature capture methods based on deep learning are also spatial resolution approaches based on imaging results. Compared with other sequencing-based omics methods, radiomics has exhibited a more promising clinical impact when combined with a standard exam, such as CA 19-9, or pathological results for PDAC patients and is mostly under evaluation in clinical studies [139]. Recently, Chinese researchers developed a deep learning framework named Pancreatic Cancer Detection with AI (PANDA) that evaluated the robustness of this model in a multicenter study [140]. Radiomics and pathology results were also integrated.

Future research will likely focus more on the spatial distribution of different cell types in the TME, such as in hypoxic, inflammatory, or vascular-rich areas, thereby enabling a more effective selection of treatment combinations.

### Reutilization of bulk data via deconvolution and generative models

Clinical patient samples are valuable, but a side effect of rapid technological advancements in recent years is the lack of sufficient single-cell sequencing samples and the high cost of sequencing. Moreover, many previous studies have generated a considerable amount of bulk-level RNA-seq and whole-exome sequencing data. Therefore, how to utilize these precious samples effectively and analyze previous cohorts has become a critical issue for rapidly advancing integrated analyses. A promising field involves the use of deep learning-based deconvolution methods and generative models (Fig. 4).

One of the most well-known applications of deconvolution algorithms is related to algorithms for immune cell infiltration. To date, software packages such as CIBERSORT [141] and TIMER [142] have been developed that can deduce the proportions of infiltrating immune cells from bulk RNA-seq data, although the results produced with these packages can vary greatly. The conclusions drawn from different algorithms in TIMER can sometimes be completely contradictory. Nevertheless, these algorithms have provided preliminary ideas for many studies, which can subsequently be validated through actual associative verification at the tissue level.

In addition to immune cell infiltration, software packages such as BayesPrism [143] and Scaden [144] can suggest prior models by learning the specificity of expression profiles from scRNA-seq data, thereby estimating the composition of cell types and the expression levels of cell type-specific genes in bulk RNA-seq data. However, these results are posterior distributions, meaning that the analysis highly depends on the stability of the input scRNA-seq data. For some rare cell types and cells with unique expression profiles, such as tumor cells, stable results are often challenging to achieve. For instance, normal epithelial cells may be incorrectly classified as tumor epithelial cells. Comparative studies suggest that BayesPrism performs particularly well when analyzing granular immune cell lineages [145].

In addition to simply analyzing the proportion of each cell type, numerous software packages based on deep learning generative pretrained models, such as scGPT [146], have been developed. These models are trained on large-scale single-cell biological data to effectively distill key genes and biological insights and support transfer learning, exhibiting good performance across various downstream tasks. Utilizing these models enables better integration of previous bulk sequencing results, combining sequencing outcomes and clinical information to achieve a comprehensive integrated analysis.

In conclusion, immunosuppressive signals from immune and stromal cells educate almost all immune cells, regulating the immune response in the PDAC TME, and numerous potential therapeutic strategies targeting these components have been developed. However, most components exhibit strong heterogeneity, and their functions and mechanisms remain unclear, especially for cells such as neutrophils, which are less amenable to detection using conventional sequencing techniques. We summarized key components within the TME at the single-cell level and proposed two feasible strategies for fully integrating the analysis of the TME, aiming to elucidate the precise relationships and mechanisms of action of these components in PDAC initiation, progression, and metastasis, thereby unraveling the enigma of PDAC.

## Data Availability

No datasets were generated or analysed during the current study.
